# ‘A ripple effect of learning’: evaluating intraprofessional workplace learning in postgraduate medical education through collective evaluation

**DOI:** 10.1186/s12909-026-09777-5

**Published:** 2026-06-23

**Authors:** M. van der Ven, D. Van Asselt, N. Looman, C. Fluit, W. Kuijer-Siebelink

**Affiliations:** 1 Geriatric Medicine Department, Radboud University Medical Center, Geert Grooteplein Zuid 10, Nijmegen, 6525 GA the Netherlands; 2 Primary & Community Care Department, Radboud University Medical Center, Nijmegen, the Netherlands; 3 Department of Research on Learning and Education, Radboud University Medical Center Health Academy, Nijmegen, the Netherlands; 4 School of Education, HAN University of Applied Sciences, Nijmegen, the Netherlands

**Keywords:** Intraprofessional collaboration, Primary care, Secondary care, Medical education, Collective evaluation

## Abstract

**Background:**

Acquiring competencies in intraprofessional collaboration (IntraPC) across the primary care (PC)–secondary care (SC) interface is essential to prevent adverse outcomes in the increasingly complex care for older adults. The workplace-based learning intervention Secondary Primary care Intraprofessional Collaboration Education (SPICE) was developed to foster IntraPC learning among co-located PC and SC residents during a geriatric rotation in the Netherlands. Understanding how learning through SPICE translates into clinical practice remains challenging and may require innovative evaluation methods. This study explored the longer-term effects of SPICE using a new collective evaluation approach.

**Methods:**

We used the effects forum, a collective evaluation method originating from applied social sciences that combines timeline analysis, mixed focus groups, and knowledge co-construction. PC and SC residents, supervisors, and patients collaboratively constructed a timeline of IntraPC learning moments during SPICE and the following six months. Participants discussed how and why these moments influenced their collaborative behavior and clinical practice. Data were analyzed using thematic analysis.

**Results:**

Seventeen participants took part in two distinct effects forums. Residents reported gaining insight into patients’ care continuum and developing greater mutual understanding across PC and SC. This facilitated more proactive handovers, which could improve continuity across care settings. Residents also transferred IntraPC learning into new contexts through reflection and dialogue, creating a ripple effect of learning that extending beyond the initial intervention.

**Conclusion:**

Workplace-based IntraPC learning can lead to meaningful changes in collaborative behavior and clinical practice. By capturing shared experiences over time, the effects forum provided insight into how learning translated into practice and supported ongoing reflection. Collective evaluation offers a valuable approach to understanding the impact of workplace learning interventions in complex healthcare settings, with potential implications for further improving continuity and patient safety through IntraPC learning across the PC-SC interface.

**Supplementary Information:**

The online version contains supplementary material available at https://doi.org/10.1186/s12909-026-09777-5.

## Highlights

IntraPC workplace learning can contribute to improvements in collaborative behavior.

IntraPC workplace learning can contribute to increased insight in the care continuum of a patient.

Collective evaluation is valuable both for deepening learning and exploring effects of workplace learning interventions.

**Supplementary Information:**

The online version contains supplementary material available at https://doi.org/10.1186/s12909-026-09777-5.

## Introduction

Multimorbid older adult patients have complex and dynamic care needs that may prompt multiple care transitions between different specialty physicians, including physicians working in primary care (PC) and secondary care (SC) settings [1–3]. Inadequate collaboration between PC and SC physicians during these care transitions is associated with fragmentation of care and an increased risk of adverse outcomes [4–7]. To prevent adverse outcomes, it is essential to have effective Intraprofessional Collaboration (IntraPC) that entails coordinated and integrated care across medical specialties guided by one integrated treatment plan [8]. Therefore, learning adequate IntraPC across the PC-SC interface is essential in the complex care for multimorbid patients [4, 5]. Developing IntraPC competencies requires dedicated learning interventions, which current postgraduate training curricula often lack [9]. Developing new educational interventions to facilitate IntraPC learning, therefore, is needed [10].

In an effort to develop new IntraPC educational interventions, the innovative workplace-based learning intervention called Secondary-Primary care Intraprofessional Collaboration Education (SPICE) was designed and implemented in the postgraduate curriculum of the geriatric department in a university hospital in the Netherlands. SPICE is a multifaceted workplace-based learning intervention in which co-located residents from PC and SC postgraduate programs doing a geriatric medicine rotation learn from, with and about each other to foster IntraPC learning across the PC-SC interface. SPICE consists of four formal learning activities and two workplace learning activities that provide residents with opportunities for open dialogue on IntraPC across the PC-SC interface (see appendix 1: SPICE program).

Workplace learning in postgraduate medical training can be understood as a form of situated learning, which is characterized by the fact that learning outcomes and mechanisms are shaped by the context in which learning occurs [11, 12]. In this setting, knowledge and skills are not acquired in isolation but are embedded within authentic practice, social interactions, and real patient care activities [12, 13]. These workplace contexts are characterized by multiple interacting elements that shape clinical decision-making under time pressure, where learning is often implicit and embedded in daily practice [14].

Currently, IntraPC workplace learning in postgraduate training largely occurs through daily interactions between residents from different specialties or between residents and their supervisors [9, 12, 15]. Within these interactions—both across specialties and between residents and supervisors—power dynamics play a significant role [16]. Hierarchies, professional identities, and underlying assumptions can shape participation, communication, and collaboration, and may at times constrain IntraPC [16]. These dynamics are particularly present in interactions across the PC–SC interface [9, 16].

In the design of the SPICE intervention, explicit attention was therefore paid to how contextual and relational factors, including power dynamics, influence both work and learning. SPICE creates a structured space for residents from PC and SC backgrounds to engage in dialogue about real-world collaborative experiences, in which they collectively explore how contextual factors, including power, have shaped their interactions. In doing so, the intervention aims to render these often implicit processes visible and open to reflection.

Because learning in this setting is inherently situated and context-dependent, the evaluation of learning interventions requires an approach that does justice to this complexity. A rich, contextually grounded evaluation makes it possible to capture the interplay between the intervention, the actors involved, and the environment, thereby offering deeper insight into how learning outcomes are constructed. Subsequently, a rigorous evaluation of the effects of IntraPC workplace-based learning interventions on clinical practice is essential, but inherently complex [17]. Initial evaluations of SPICE, using in-depth and focus group interviews during and immediately after participation, indicated promising effects in terms of perceived learning and behavioral intentions. How these results translate into effects in clinical practice, however, remains unclear.

Effects on clinical practice may include improved patient outcomes, improved patient and/or caregiver satisfaction or improved collaborative behavior resulting in more coordinated and integrated care [18]. Traditional evaluation methods may fall short of exploring the effects of workplace-based learning interventions on clinical practice [17]. Quantitative evaluation methods, such as questionnaires or patient-related outcome measures like mortality or hospital readmissions, are subject to a multitude of confounding factors within the complexity of clinical practice [1–3]. These confounders may include recall bias, inadequate therapy adherence, informal caregiver burden or interacting comorbidities, which are particularly apparent in the care for patient groups such as multimorbid older adults [1–3]. As these confounders may obscure whether observed changes in clinical practice can be attributed to a specific learning intervention, this complicates the use of quantitative methods in understanding the effects of workplace-based learning interventions on clinical practice [1–3].

Qualitative methods aimed at exploring the effects of workplace-based interventions on clinical practice are promising, yet they require an approach that adequately accounts for the complexity of the clinical environment. Workplace-based interventions are often multifaceted, making it challenging to capture the full scope effects of the intervention through, for example, individual interviews or observations conducted immediately after formal learning activities. In addition, it can be difficult to identify what informal moments in practice may also have contributed to IntraPC learning. As a result, it may prove difficult to disentangle what components of learning interventions drive meaningful changes in clinical practice [17, 19].

Despite these challenges, evaluating the effects of IntraPC learning interventions such as SPICE remains vital, especially in times of increasing scarcity of personnel and financial resources [20, 21]. New learning activities are also often layered onto existing curricula, further expanding their content [22]. Consequently, learners in the workplace face rising workload pressures, which have been linked to increased incidence of burnout [22]. Careful evaluation of new workplace-based learning interventions, therefore, is essential, not only to ensure their effectiveness and feasibility, but also to prevent them from inadvertently contributing to excessive workload while ensuring they deliver meaningful effects. These considerations highlight the need for innovative evaluation approaches that can capture different perspectives, identify influential components and underlying learning mechanisms and clarify longer-term effects on clinical practice.

As workplace-based learning is inherently a social process amongst stakeholders, an evaluative approach that mirrors this collective and social learning may be promising for exploring effects of workplace-based learning interventions in clinical practice [23, 24]. In this study, we explore the longer-term effects of SPICE on residents’ collaborative behaviors and clinical practice, six months after completing the program, using a collective evaluation method called ‘the effects forum’. 

**Table:** Box 1: Contextual information of the Dutch medical education trajectory, workplace setting and SPICE interventions

In the Netherlands, medical education consists of undergraduate medical training (bachelor and master level) followed by postgraduate specialty training. While undergraduate training combines formal education with clinical internships, postgraduate training is predominantly workplace-based.
During postgraduate training, physicians in training (residents) participate in clinical rotations within and outside their specialty. Their training is formally overseen by a program director, while their day-to-day clinical work and learning are supervised by different clinical supervisors in the workplace. These supervisors are medical specialists tasked with both overseeing effective task execution and patient safety as well as facilitating workplace learning. Workplace learning is therefore central and characterized by learning through participation in clinical practice. In addition, weekly-scheduled teaching hours provide opportunities for more formal learning activities alongside clinical work.
Within the Dutch healthcare system, PC physicians act as gatekeepers to SC, making collaboration across this interface a routine yet complex aspect of clinical practice. This is particularly relevant in geriatric care, where patients often present with multimorbidity and require coordinated input from multiple specialties.
Against this background, a team of professionals from diverse clinical, educational, and research backgrounds developed the Secondary Primary Care Intraprofessional Collaboration Education (SPICE) program. SPICE is a program integrated into clinical rotations, consisting of a combination of formal IntraPC learning activities and workplace-based learning tasks. The program builds on the co-location of residents from PC and SC training programs and encourages reflective dialogue around real-world PC–SC interactions. Within SPICE activities, a moderator prompts residents to exchange perspectives, reflect on key collaborative moments, and discuss how contextual factors, such as power dynamics and professional assumptions, may influence IntraPC.

## Methods

To evaluate the longer term effects of SPICE, we used a constructivist approach [25]. To appropriately evaluate a complex intervention within a tertiary care context, a methodological approach that acknowledges and accommodates complexity and dynamics is essential. Therefore, we chose ‘the effects forum’, which is a collective evaluation method developed in the applied social sciences field in the Netherlands [26]. Collective evaluation is an approach in which a group of stakeholders jointly reflect on an intervention [27, 28]. Rather than relying on individual assessments or evaluations, this method uses shared experiences and dialogue. Its aim is to collect multiple perspectives and collaboratively construct meaning regarding the effects of the intervention. 

### Effects forum

The effects forum is a group discussion format, in which all stakeholders of the learning intervention are represented. Stakeholders collectively evaluate the effects of an intervention on the personal, interpersonal and system level using a timeline. The effects forum uses elements of (1) focus group interviewing, (2) co-construction of knowledge and (3) timeline analysis [26].

1. Focus groups are a method of data collection that gathers opinions, insights and ideas from participants, who can then collaboratively generate deeper insights into their experiences with an intervention [29]. By employing a heterogeneous group (in our case comprising PC and SC residents, supervisors and patients), participants can reinforce, contrast or challenge each other’s narratives [27]. This dynamic fosters a more comprehensive understanding and allows individual findings to be contextualized and transferred to other settings.
2. Co-construction of knowledge is a concept that has been increasingly embraced in qualitative research that aligns well with our constructivist epistemology. It enhances data quality by incorporating multiple perspectives and encouraging examination of the data from various angles [30, 31]. This is particularly valuable to equalize voices in order to gain broad insight into issues impacting SPICE outcomes, including potential power dynamics. In the effects forum, co-construction is stimulated by including all stakeholders in the same focus group and by appointing one participant per effects forum session as the ‘effects master’. The effects master is a participant who helps the other participants to become aware of any effects mentioned during the effects forum. The effects master is empowered to interrupt the discussion to prompt deeper exploration of any effects mentioned. The effects master writes memos during the effects forum to summarize the uncovered effects and stratifies them into categories of personal effects, interpersonal effects or systems effects. Each participant can volunteer for the position of effects master.
3. Timeline analysis serves as a valuable tool as it enables identification and exploration of significant learning moments in the workplace that extend beyond those formally addressed during the intervention [32]. By using this approach, it is possible to uncover insightful learning opportunities that might be missed when relying solely on direct questioning about outcomes of formal workplace-based learning activities [32]. Moreover, when participants are asked to recall their experiences, they may easily forget important elements, particularly those that occurred further in the past. By collaboratively reconstructing events along a timeline, participants are supported in retrieving memories. This allows for richer data collection and a more comprehensive understanding of the intervention’s sustained effects while limiting recall bias.

**Fig. 1:**
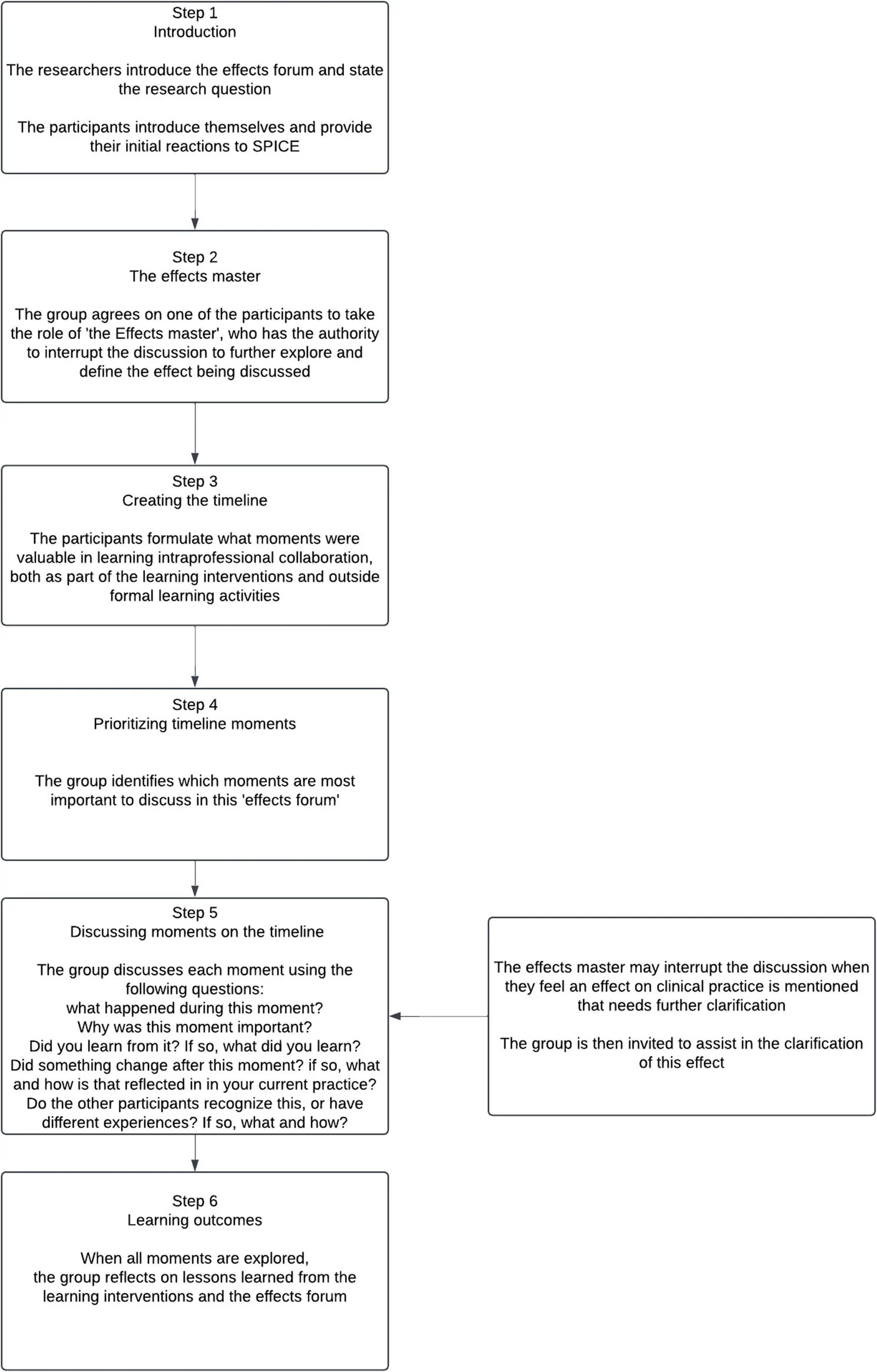
The data-generation process during the effects forum

The process of data generation during the effects forum is depicted in Fig. 1. In our study, the effects forum began with step 1: an introduction to the topic, in which participants could share their initial reactions to IntraPC learning and SPICE. In this introduction, we asked participants to introduce themselves including their professional backgrounds, and to express what they felt was important in IntraPC learning across the PC-SC interface. This was followed by step 2: agreeing who would participate as effects master. In step 3, the timeline was introduced.The learning activities that were part of SPICE had already been placed. Participants were then invited to add moments that influenced their IntraPC learning across the PC-SC interface. Participants were encouraged to add actions and interactions that mattered to them regarding IntraPC learning in the workplace. Subsequently, in step 4, the group collectively prioritized which of these moments were most important to elaborate upon. In step 5, each selected moment was discussed in turn, guided by the following questions:

- What happened during this moment?
- Why was this moment important?
- Did you learn from it? If so, what did you learn?
- Did something change after this moment? If so, what and how is this reflected in your current practice?
- Do other participants recognize this, or have different experiences? If so, what and how?

In this way, all moments were ’unpacked’. Lastly, in step 6 participants were asked to reflect on their key takeaway from SPICE or from the dialogue during the effects forum itself. 

### Participants and setting

Given the collective nature of the evaluation, purposive sampling was essential to ensure the inclusion of relevant perspectives. All participants in the evaluation process were regarded as equal contributors, with each having an equal voice in the dialogue. The participants reflected representatives from each stakeholder group. Stakeholders in this learning intervention included residents, supervisors and patients, all of whom were purposefully selected to reflect the diversity of perspectives involved.

The residents were enrolled in PC or SC postgraduate training programs and were doing or had done a rotation in geriatric medicine. These residents were either enrolled in the PC General Practice and Elderly Care programs, or the SC Geriatric or Internal Medicine programs (Table 1: medical specialties in the Netherlands). To evaluate the longer-term effects of SPICE for these residents, we invited residents that had finished their SPICE activities at least six months ago. Therefore, we recruited residents that had returned to primary care settings and had moved to another secondary care rotation in another clinical or research department, which provided us with the opportunity to explore how skills acquired in SPICE were transferred to their new placement.

**Table 1:** Overview medical specialties in the Netherlands

General practitioner (GP)	PC doctor ‘working in the frontline of a healthcare system’, taking the initial steps to provide care for any health problem(s) that patients may have, including prevention, diagnosis, cure, care, and palliation.
Elderly care physician	PC doctor working in long-term care for older adults and chronic patients, mostly in a nursing home. In the Netherlands this is a PC specialty.
Geriatric medicine	Doctor working in acute care for frail older adults in a hospital setting. They work includes outpatient consultation as well as inpatient clinical practice. In the Netherlands, this is a SC specialty
Primary care (PC-setting	The first, community-based medical care. The gatekeeping role of PC doctors makes them responsible for adequate referral of patients to hospital care.
Primary care resident (PC resident)	PC residents are in training to become GPs or elderly care physicians. Both PC postgraduate training programs involve a 3-year competency‐based program. The PC residents provide patient care in the PC setting during their first and third year. During their second year, PC residents undertake other placements, amongst which is a hospital of 6‐9 months.
Medical specialist	Doctor providing specialist medical care. Mostly offered in hospital settings, where both inpatient and outpatient clinics are combined. In the Netherlands, geriatricians are medical specialists working in SC.
Secondary care residents (SC residents)	Doctor in training to become a medical specialist. The postgraduate medical specialist training involves a 4-6‐year competency‐based program. The medical specialist residents provide patient care in a hospital, or other secondary care setting (SC).

The supervisors were geriatricians that were responsible for overseeing the clinical work of the residents during their geriatric medicine rotation and were responsible for workplace learning during these rotations.

Prior to the effects forum, participating residents were asked whether they had encountered a patient during their rotation in geriatric medicine who might be suitable for inclusion. Particularly patients with no or limited cognitive impairment and preferable where IntraPC challenges were a part of the assessment were deemed eligible. So, the patients were older adults that had visited the geriatric medicine department of the university hospital in which SPICE was implemented. These patients had received care from one or more residents present in the effects forum while they were participating in SPICE activities during their geriatric medicine rotation in the department.

The study setting was a geriatric medicine department in an academic hospital in the Netherlands. This department includes an out-patient consultation service, an in-patient acute care ward, an attending geriatric resident in the emergency department and a geriatric liaison team for in-patients. Its staff consists of nine geriatricians, two internists and a varying group of residents from different primary and secondary care specialty training programs. For several years now, IntraPC learning has been a topic of research and resident and supervisor training at this department. 

### Data generation

We performed two effects forums; the first was held in March 2022 and the second in January 2024. The participating residents had enrolled in SPICE the year before taking part in the effects forum and had finished at least six months ago. The effects forums were held in a hybrid format: participants could participate either live or through video-conference call to facilitate participation for residents that had moved on to PC and SC contexts in different parts of the country and for less mobile patients. The effects forums lasted two hours each and were moderated by two researchers: a geriatrician (MV) and a psychologist (NL). The first moderator was the discussion leader, and the second paid attention to participation of the participants in the video conference. A third researcher, an educational researcher (WKS), observed interactions and behavior during the session and supported the effects master in memoing. The interview guide was created using insights from reflective interviewing [33]. (see appendix 2: interview guide)

### Data analysis

The data consisted of the effects master memos, the timelines and the audio recordings of the effect forum sessions that were transcribed verbatim. The data were analyzed through thematic analysis in six iterative steps (see below) [34]. This process was supported by regular research group meetings to enhance reflexivity [31]. These meetings involved collaborative reflection on codes, quotes and preliminary themes across effects forums transcripts, timelines and effects master memos. The research group, could provide their perspectives and theoretical insights in these meetings [22]. These insights could then lead to codes being reframed and/or stratified. This analytic process resulted in three overarching themes describing the long-term effects of the SPICE program as perceived and discussed by stakeholders. 

#### Step 1: timeline and memo analysis

The timeline and the effects master’s memos were reviewed. Memos were read and re-read and the researchers checked the stratification into personal, interpersonal and system level effects provided by the effects master. The timelines consisted of instances that participants had experienced as moments of IntraPC. We developed code templates based on the learning moments mapped on the timeline, which we could then use for the initial coding of the transcripts. 

#### Step 2: within-session analysis

Verbatim transcripts of the effects forums were analyzed combining template analysis and inductive coding using Atlas.ti. Two researchers (MV and WKS) independently conducted template and open coding, meeting regularly to discuss coding decisions, resolve discrepancies and ensure consistency. They used the code templates created from the learning moments on the timeline to identify the selected IntraPC learning moments and the corresponding sections in the discussions. Through open coding, they then explored learning outcomes, changes in clinical practice and professional behavior in each of the selected learning moments. 

#### Step 3: cross-session analysis

**Fig. 2:**
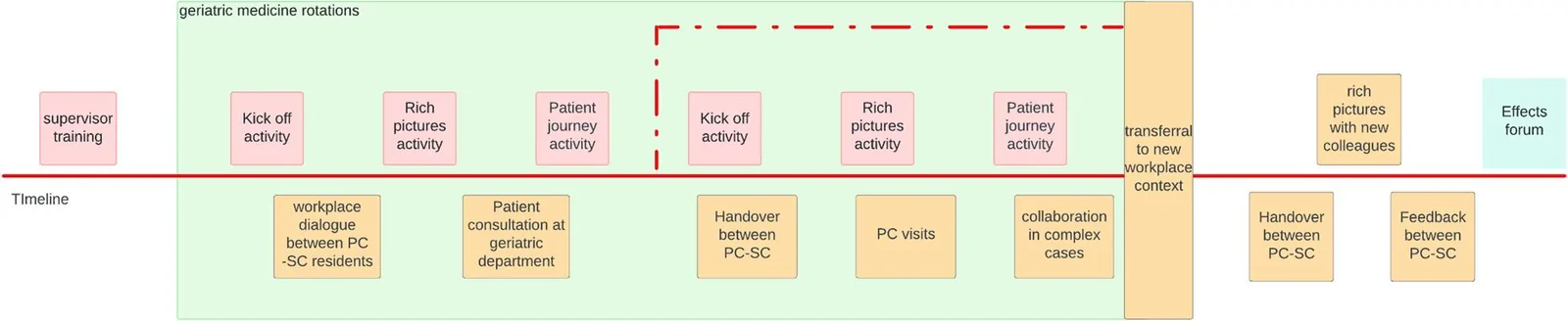
Timeline with meaningful moments for IntraPC learning. During the effects forum, participants identified meaningful moments during the last months that had contributed to learning IntraPC. This timeline guided the discussion between participants in the focus groups. The red line is the timeline, the dotted red line depicts how some geriatric medicine rotations take only three months, so their timeline branches of to a new workplace setting earlier. The red boxes relate to formal learning activities residents participated in during the geriatric medicine rotation. Orange boxes relate to moments in clinical practice that resulted in informal learning on IntraPC.The blue box relates to the research activity described in this paper

Template codes describing valuable IntraPC learning opportunities from both effects forums were compared and synthesized. Similar learning moments were merged and mapped onto one integrated timeline (Fig. 2).

#### Step 4: combining IntraPC learning moments templates and open codes

For each learning moment on the integrated timeline, co-occurring open and template codes and associated quotes were re-read to explore learning outcomes in practice and behavior with each learning moment. This step generated a set of illustrative quotes and code groups linked to each specific learning moment on the integrated timeline. 

#### Step 5: overarching comparison across timeline, effects master memos and transcripts

The resulting code groups and quotes were then combined with the effects master memos to identify overarching preliminary themes that captured the effects observed in clinical practice.

Codes were iteratively grouped into higher-order categories, which were subsequently abstracted into preliminary themes. Throughout this process, the different data types could enrich each other. 

#### Step 6: Finalize themes

Finally, the preliminary themes were reviewed across the entire dataset, including other learning moments, in order to verify thematic saturation [35]. 

### Reflexivity

From a constructivist perspective, we assume that data are inherently shaped by contextual and relational factors [36]. Rather than viewing these context-specific characteristics as sources of bias to be minimized, we aimed to provide a rich and transparent account of how such factors potentially influence both data generation and interpretation.

A key consideration concerns the potential relational dynamics among participating patient and residents, residents and supervisor, the participants and the researchers and the effects master and the researchers:

- Patients were purposively selected from the clinical practice of participating residents, resulting in pre-existing care relationships. While this sampling strategy may have influenced participants’ willingness to express critical perspectives, it enabled patients to provide direct, experience-based feedback on residents’ collaborative behavior.
- The role of supervisors in relation to residents may also shape data generation. Supervisors inherently play a role of varying degrees in training of participating residents during the geriatric medicine rotation, which may affect residents’ willingness to voice critical reflections. To promote psychological safety, residents were selected who were no longer actively working within the studied department. Additionally, moderators deliberately framed IntraPC in terms of a result of broader contextual dynamics rather than solely an assessment of individual performance.
- The positioning of the research team may also influence data generation and interpretation. Moderator MV held multiple roles as researcher, supervisor and teacher during SPICE formal learning activities. To acknowledge potential dual role tensions, multiple moderators were involved providing the opportunity to interject during the effects forums at times where MV’s individual perspective might risk taking the upper hand. During data analysis, the research group reflected on how each individual perspective influenced data analysis. Furthermore, moderator NL has specific expertise in observing power dynamics and was attentive to participants’ ability to speak freely. Furthermore, particularly in the second effects forum, participants were explicitly invited to reflect on unintended or ‘negative’ outcomes of the SPICE intervention.
- The role of the effects master may also shape the data. As this role was fulfilled by a participant, this dual position may have limited their ability to fully articulate their own perspective. They may also feel a barrier to interrupt the discussion. To support this role, moderator WKS was appointed as a second effects master. During the effects forum, the moderators explicitly create space for the effects master to contribute their own perspective or intervene to explore effects in more detail.

Importantly, the research team comprised members with diverse clinical, educational and research backgrounds, enabling a team-based approach to data collection and analysis. This diversity supported ongoing critical reflection on emerging interpretations and facilitated the explicit consideration of different perspectives and assumptions, thereby strengthening the credibility and robustness of the analysis. 

### Ethical considerations

This study was performed in accordance with the Helsinki Declaration. Ethical approval for this study has been obtained from the Ethical Review Board of the Netherlands Association for Medical Education (NVMO-ERB) with protocol number 2020.7.3.

Written informed consent for participation was obtained before participation in the effects forum. Participants gave consent for publication of their pseudonymized data.

## Results

Two effects forums were held with a total of 17 participants, including 12 residents, 2 supervisors and 3 patients. The characteristics of the participants in each effects forum are depicted in Table 2. In both effects forums a resident voluntarily came forward as effects master, a SC resident in the first effects forum and a PC resident in the second. The other participants expressed agreement with this allocation of roles.

**Table 2:** Participant characteristics

N of participants	17
Residents	12
Supervisors	2
Patients	3
Participant age (range)	
Residents	28 – 42
Supervisors	35 - 39
Patients	69 – 74
Sex (F/M)	12/5
Residents’ specialty backgrounds	
Primary care	7
- General Practice	3
- Elderly Care	4
Secondary care	5
- Geriatric Medicine	3
- Internal Medicine	2

The moments identified by participants to be unpacked, included the SPICE formal learning activities, dialogues amongst residents during their geriatric medicine rotations, collaboration in complex patient cases, dialogues between PC-SC during clinical practice and interactions with colleagues after transferring to new rotation contexts. We visualized the valuable moments of both effects forums in one integrated timeline (Fig. 2). Our thematic analysis resulted in the following overarching themes: insight into patients’ care continuum, improved mutual understanding and actionable awareness and the ripple effect of IntraPC learning. 

### Insight into patients’ care continuum

In SPICE formal learning activities, residents exchanged perspectives and discussed how clinical practice differed across the PC and SC care contexts, and they could provide feedback on their collaboration, for example, on their handover or referral letters. The residents reported how SPICE activities improved their insight into the care continuum patients may be subjected to in their journey across care settings and how an individual resident’s work is only a small part of that. Patients noted that they often had limited insight into interactions across PC-SC that occurred. They mentioned they were not always aware of the intentions of their referring PC physician that were transferred across the care continuum, which could differ from their own intentions for SC referrals. In addition, patients noted that they often had limited insight into the collaboration between PC and SC, while expressing appreciation for greater involvement in referral.

*‘I think it is quite difficult*,* because you tell your story at the GP*,* who listens. Then the GP makes a referral*,* but you are not aware of which information reaches the specialist and you don’t know if this information aligns with your perspective on your complaints. The specialist then acts using this information the GP provided. […] I think it’s a shame*,* because you as a patient would want to add your own little note attached to the referral letter.’ - *patient.

Subsequently, residents and supervisors mentioned they observed a noticeable shift in their perspective towards patient care across the PC-SC interface: while their initial focus had predominantly been doctor-centered and hospital-based, they mentioned to become more open to a patient-centered view within the PC-SC collaboration network.

*… In my first drawing I eventually put the hospital doctor in the middle […] with the patient right next so it’s really … actually*,* I’m really doing just that*,* aren’t I*,* my patient symbolized the core but the hospital doctor was actually in the middle. Kind of striking*,* isn’t it. […] But when I’m just looking at my way of thinking*,* I think that this has changed.* - Elderly Care Resident.

*What changed*,* I think*,* was that I realized much more clearly that*,* hey*,* hang on a minute*,* we’re not in the hospital stronghold here but we’re moving into … we’re operating in a network. I knew that*,* of course*,* but I started to pay more attention to it.* - Supervisor.

Residents mentioned how insights from SPICE prompted them not only to make more phone calls for explicit handovers across PC-SC, but also to give more feedback to the physician in the other setting.

*Only last week I spoke to a surgeon on the phone. About why they had admitted a patient for analgesia without mentioning the analgesia plan in their [discharge] letter. Yeah*,* I would have hesitated to do so before SPICE for*,* well*,* you don’t really want to bother a surgeon. But I felt this mattered so much to the patient that I did make the call and SPICE has really helped me to do so.* - Elderly Care Resident.

One of the PC residents mentioned that the insights on IntraPC had made them reflect on the current practices for people in crisis. In their opinion, current interactions between the general practitioner, elderly care specialist and geriatrician for patients in care crisis are often difficult, impacted by competing interests and logistical barriers. They felt current practice for patients in care crisis could benefit from more effective intraprofessional deliberation. SPICE had given her new ideas for discussions among the three involved specialists to enable swift, efficient and limited hospital diagnostic assessment followed by a transition to nursing home care for older adult patients during a care crisis. This idea resonated with the supervisor.

*I realized it’s a pity we don’t have a consultation about people in crisis*,* amongst the three of us. So the Elderly Care specialist on duty*,* the GP and the geriatrician*,* initiated by the one*,* of course*,* who’s having doubts*,* I suppose. Why don’t the three of us meet up briefly to discuss the case? That would be a nice goal for the future. So that was the upshot*,* that I thought … I’m still … that’s still going through my mind. That I’m thinking: why don’t we do this in the future.* - Elderly Care Resident.

*Well I’m up against this every week*,* so I’d love to discuss this with you to come up with a solution. Because I think we can develop a new care model for this within a week.* - Supervisor.

One patient added that the Geriatric Medicine resident in charge had contacted other members of his treatment team, after which they noticed the other physicians in their care network approach them differently. In their opinion, their other treating physician now had a more integrated understanding of the interactions of their multiple medical conditions. The patient felt this improved insight into their interacting conditions led to a more patient centred approach in communication and in their treatment.

*What I noticed is that the [other physician’s] tone had completely changed. Before that*,* they tended to patronize me*,* you know: ‘pull yourself together and something’s always bothering everyone.’ After that*,* though*,* I was suddenly taken very seriously indeed*,* I noticed*,* and I still am.* - Patient.

### Improved mutual understanding and actionable awareness

In the effects forums, participants discussed how encounters between PC-SC physicians had contributed to their improved understanding of different ways of working. Moments mentioned on the timeline that had prompted IntraPC learning tended to be dialogues with a physician from the other work setting. This included dialogues with fellow residents they encountered in the geriatric ward, encounters with colleagues during daily handovers across the PC-SC interface and (particularly for SC residents) during PC visits as part of SPICE.

*‘The conversations you have with each other*,* that while we all do a rotation at geriatric medicine*,* while you do shared assignments*,* but also just*,* the conversations you have in between*,* about what is it you [SC resident] do [.]. What is my perspective as elderly care physician but also what is your perspective as internist and how you share these [perspectives]. …This makes it easier to understand what the other wants and how the other thinks’* – resident elderly care.

An SC resident visited a PC setting as part of SPICE, where observations of the work of PC residents provided her with insight into the work context of the nursing home.

*Yes*,* there was a very specific moment [during a PC visit] that was most telling. I was talking to her when we were called about someone who was very short of breath. So the two of us went over there. So she*,* an Elderly Care specialist in training*,* she looked at this lady*,* put a hand on her shoulder and said to the nurse: ‘Just give her Lasix 80 mg’ and then we left. And I felt an urgent need to sort of take her history*,* to ask her whether she felt any chest pain*,* or just to listen [to her lungs] or that sort of thing*,* so I told her and then she said: ‘Oh no*,* yes*,* no*,* she’s just a little overwhelmed by it all so I’m just giving her this.’ And half an hour later*,* this lady was sitting in her chair chatting away*,* everything back to normal. So I … so this was a very concrete moment for me to witness a very different way of working.* – Internal Medicine Resident.

During daily interactions between PC and SC, residents explored each other’s contexts, values and ways of thinking. The participating residents mentioned that this exploration enhanced their mutual understanding. This improved understanding encouraged them to improve their collaborative behavior and clinical practice. Furthermore, participants stated that they had become more aware of the impact their actions could have on professionals in the other care setting. They mentioned they felt this improved understanding was also reflected in more respect towards each other’s profession.

*So really making sure your handover is even better*,* with medication specified. And making sure your patients and their relatives are informed about the treatment plan you’re proposing. We had very concrete discussions with the Elderly Care specialist about advance care plans limiting renewed hospital admissions. He said it happens sometimes that patients or their relatives are not aware of this [decisions made during hospital admission] at all or that it hasn’t even been discussed with them*,* so that they haven’t just missed it somehow but that it actually hadn’t been discussed at all. If you fail to do so*,* this really causes a huge problem for the Elderly Care specialist. I’m not saying I’ve done things in that way before*,* but it just helps to make you more aware of that sort of thing.* - Internal Medicine Resident.

For some residents, this improved understanding prompted them to reflect on their clinical practice and to explore how their actions could be better aligned with physicians in other contexts. We have called this ‘actionable awareness’: the process by which residents developed a deeper understanding of each other’s professional contexts, routines and cognitive frameworks, which in turn prompted them to reflect on how their own actions influenced cross-settings care. Importantly, this reflection also motivated residents to adapt their practices in ways that promoted better integration and coordination across settings.

The PC residents mentioned that this actionable awareness encouraged them to formulate referral questions in a more concise and direct manner. One resident reported that their improved referral question led to more concise responses from SC colleagues. For example, a PC resident referred a patient to neurology for further diagnostics, not because they strongly suspected a brain tumor as the cause of the patient’s headaches, but because the patient could not be reassured in primary care. In response, the neurologist provided a more concise answer than the resident had experienced in previous referrals.

*They tend to deal with my question more specifically. […] I’ve got an example. […] I had a patient with a headache who thought: ‘I’ve got a brain tumor.’ And I was unable to reassure them sufficiently so I referred they*,* saying: ‘Low probability but fail to reassure patient. Is there any likelihood of there being a brain tumor*,* yes or no. Please present you findings.’ And then they [hospital specialist] said very specifically: ‘Headache but no brain tumor. Continue treatment plan.’ So it’s dealt with more specifically whereas normally I might have said: ‘Headache analysis please.’ And then they would have said: ‘Headache no alarming symptoms*,* watchful waiting.’* - General Practice Resident.

SC residents, on the other hand, mentioned to have learned to be more explicit in discussing the expectations that the referring PC physicians and their patients might have of SC assessment and treatment. If the referral question was clearer, they felt their SC diagnostics and treatment plan could be better tailored to the needs of the patient and the referring PC physician.

*Yes*,* but I do think it really helps if the other person is also more aware of what they can expect from a colleague in the hospital. Or the other way around*,* that you learn to ask a colleague specifically: ‘But when I’m hearing X*,* I’m thinking Y*,* and why do you think this is not the case? Or: So what is really your question?’ So when everyone is more SPICE-trained*,* you learn to question each other in that way.* - Supervisor.

### The ripple effect of IntraPC learning

On the timeline, residents added that after they had rotated from their Geriatric Medicine placement to their next specialty placement, which could entail going back to their own specialty’s workplace or to a new out-of-specialty rotation or a research placement, they experienced meaningful IntraPC learning moments in their new context. Residents mentioned that, in the practice of this new placement, they were able to use the reflection and dialogue skills they had acquired in SPICE. Dialogues with colleagues in their new workplace, prompted reflection through comparing and contrasting PC and SC practices.

*‘… recognition and similarity*,* […] a lot more that difference actually. And also alignment. That is the motivation to keep on looking for contact and alignment during [intraprofessional] deliberation.’* – resident internal medicine.

Residents mentioned this continued use of reflective dialogue caused them to transfer IntraPC learning from the SPICE activities in the geriatric department to their new workplace. One participant described this as the ripple effect of IntraPC learning.

*Gotta keep talking about things. When you’re in charge of patients at A&E: that’s a pretty vulnerable man really. Turns out to be a patient for Internal Medicine*,* but I could have seen him in Geriatrics during my Geriatrics rotation. In terms of your treatment plan*,* it might be interesting to compare two cases*,* you know: two patients arriving at A&E independently*,* compare and contrast both treatment plans. Not necessarily with one being any better or worse than the other one*,* but it’s kind of interesting to see how different perspectives can be.* - Internal Medicine Resident.

One of those meaningful learning moments was when a nursing home resident shared that they had arranged a Rich Pictures session for their new PC colleagues.

*After I had arrived in my new rotation*,* I also did a mini SPICE moment for them using a rich picture*,* so I delivered new knowledge but I also obtained new knowledge. So I explored my SPICE knowledge in greater detail in the care home setting.* - Elderly Care Resident.

As lessons learned in SPICE were shared in new work contexts, residents noted how the colleagues in their new workplace became increasingly motivated to engage in more explicit IntraPC across the PC-SC interface, particularly when handovers were positively received by colleagues in the other setting.

*Yes*,* I think it’s what I just said*,* that I see it happening in my own work. You see it spreading as a ripple effect*,* and I’m pleased to hear that [Resident 12] and [Resident 9] are also referring to it*,* and they may infect their colleagues*,* so it all becomes more normal. And*,* as [Resident 12] observed*,* it also helps because you carry it on to the next one. I also find it easier when I’m talking to a surgeon on the phone: Is it a straightforward answer you want from me? What exactly is your question? And perhaps you’re making light of it*,* you know*,* trying to find common ground in your collaboration with them. Just take your time in a couple of sentences.* - Supervisor.

As the participants noticed their awareness of the importance of handovers between PC and SC had increased, they initiated more frequent and detailed handovers by phone in their new context. While in their opinion, this contributed to improved continuity of care, it also led to an increase in workload. Moreover, when residents transitioned to new clinical environments where SPICE or IntraPC learning was not common practice, their efforts to engage in more comprehensive handovers were sometimes met with confusion or frustration from other physicians, who at times perceived these handovers as unnecessarily time-consuming.

*So sometimes it’s discouraging because you know that in other regions you will just have to carry on in the old way*,* shame*,* but the [IntraPC] knowledge is lacking there. Oh well*,* I’m positive about it anyway: it’s an opportunity to impart new knowledge*,* isn’t it.* - Geriatric Medicine Resident.

*So sometimes you may get a slap in the face from angry colleagues who’re thinking: ‘ What is it you want*,* for goodness sake?’* - Supervisor.

Participants mentioned how they felt the effects forums themselves also contributed to IntraPC learning.They were reminded of lessons learned, acquired new insights and could transfer these into their current workplace. The presence and active participation of patients was particularly well received.

*Erm yes*,* I particularly appreciated the patients being there. I’d already jotted down for myself that it’s so important to listen to their request for help and to synch up and to align expectations. It reminded me of this.* - Internal Medicine Resident.

*Today I was again made aware how important it is to keep applying the SPICE lessons in day-to-day work.* - Internal Medicine Resident.

## Discussion

This study illustrates the exploration of the effects on collaborative behavior and clinical practice of the Secondary-Primary Care Intraprofessional Collaboration Education (SPICE) program. SPICE was designed to foster intraprofessional collaboration (IntraPC) across the primary (PC)-secondary care (SC) interface. By using the effects forum, an innovative collective evaluation method, stakeholders created a shared timeline of meaningful IntraPC learning moments and co-constructed effects through dialogue. These moments included the formal SPICE learning activities. Participants reported that formal SPICE learning activities fostered an awareness of the care continuum and promoted a shift from a doctor-centered to a patient-centered perspective. Participants mentioned how they translated this improved insight into more deliberate handovers. They agreed that these handovers could be used not only to transfer clinical information but also to provide feedback on treatment plans and collaborative practice across care settings. Other valuable IntraPC learning moments were workplace dialogues between PC and SC physicians, both during and beyond SPICE activities. These dialogues were experienced to contribute to a clearer understanding of the capabilities and contexts of colleagues in another care setting and reflected on how their own behaviors influenced work in another care context. Finally, participants mentioned how, after they had transferred to their next rotation, they had experienced new important IntraPC learning moments, which they felt could lead to IntraPC lessons being transferred to their new colleagues and renewed reflection, expanding the ripple effect of IntraPC learning to other settings.

Participants described how they had experienced interactions with professionals from other care settings to provide valuable insights into PC–SC collaboration, which had prompted them to reflect and improve on their collaboration behavior. Through this collective evaluation, the importance of dialogue among stakeholders in both learning at the workplace and during evaluation became evident. During our analysis, we discovered how these findings can be understood through the lens of social constructivist learning theory, which emphasizes that learning is socially situated and co-constructed [37]. Within this framework, dialogue is not merely an exchange of information but a process in which participants co-create understanding and develop shared meaning [24]. While traditional medical education primarily focuses on biomedical knowledge acquisition [23], the SPICE module intentionally created structured opportunities for dialogue and reflection on collaborative processes across PC and SC. These social engagements in SPICE facilitated the development of reflective and collaborative competencies which are indispensable in today’s complex clinical environments, where professionals must learn how to transcend disciplinary boundaries to deliver integrated care [38].

The social constructivist nature of the collective evaluation mirrored the program’s social learning mechanisms, aligning the process of evaluation with the principles underpinning learning in SPICE. This evaluative moment, therefore, may be interpreted as an extension of the learning process, effectively reflecting and reinforcing the intended educational outcomes.

It is noteworthy that participants described translating lessons from SPICE into new clinical contexts of subsequent rotations. Previous research on workplace learning has shown that rapid socialization into the local norms of a new rotation may come at the cost of previously learned behaviors [39]. Transfer in health professions education requires learners to integrate declarative and procedural knowledge into a coherent mental model, which can be reactivated in new clinical situations [40]. Metacognition appears to play an essential role in creating mental models, enabling learners to interpret the individual demands of the new situation and regulate their thinking and working accordingly [41].

Our findings question the possibility that SPICE may have supported the development of a mental model for IntraPC. A shared mental model is a common representation of tasks, roles and strategies that enable team members to anticipate each other’s actions and coordinate effectively [27]. Both SPICE and this evaluation emphasized metacognitive processes through collective reflection on IntraPC across the PC-SC interface. Having been provided with different formats for structured collective reflection, participants from PC and SC might have developed a shared mental model of IntraPC during SPICE. Importantly, if this model was indeed co-constructed by stakeholders from PC and SC, it could be used as both a cognitive resource and a shared framework to strengthen coordination and mutual understanding during IntraPC across the PC-SC interface in diverse clinical settings. Shared mental models are known to facilitate coordinated and integrated teamwork in clinical practice and promote the acquisition of IntraPC competencies during workplace learning, and they can be useful, therefore, in diverse clinical settings in subsequent rotations [14, 42]. It would be interesting for future research to explore whether SPICE activities indeed contribute to clearly defined mental models of IntraPC.

Subsequently, Participants reported that, upon moving to new rotations, they encountered authentic clinical situations that enabled them to deepen their IntraPC learning. From an interpretive perspective, such experiences may support the ongoing development and refinement of residents’ mental models related to IntraPC. Through encountering new situations, residents may test and adapt these understandings, which may contribute to a growing sense of confidence in navigating PC–SC interactions. In turn, participants suggested that this evolving understanding enabled them to apply and share their perspectives with colleagues in new settings, potentially contributing to a broader ripple effect of intraprofessional learning. 

### Implications

Our findings provide suggestions for educational practice, research and policy. Participants mentioned how the SPICE intervention helped them to make intentions for improved collaborative behavior across the PC-SC interface. Moreover, residents mentioned how they transferred their new insights intonew contexts. The combination of formal activities that provide shared reflective moments and practical, workplace-based learning activities enabled these residents to engage in IntraPC learning in real clinical settings. This indicates the potential value of incorporating dedicated IntraPC workplace-based learning in postgraduate training.

This study also explores the educational potential of evaluation research. Prior work has shown that evaluation processes can themselves become meaningful learning interventions. This potential lies in the generative moments that occur during evaluation [43]. Generative moments energize participants and create space for new insights [43]. Previous research has indicated that these generative moments often arise during social interactions where the importance of change is explicated and when lessons can be taken to new workplace settings where reflective reinterpretations occur [44, 45]. Elements that enable generative moments to occur, appeared to have been presented during SPICE and the subsequent months afterwards. This suggests generative moments may have occurred within the studied timeframe. In this sense, the collective evaluation aligned seamlessly with the social constructivist mechanisms underlying the intervention. Further research could explore whether future postgraduate education initiatives could benefit from incorporating such collective evaluations to strengthen ongoing learning across healthcare settings.

Moreover, this study suggests scientific potential of education evaluation research for exploring the effects of complex workplace-based learning interventions. This method combined multiple perspectives, possibly by enabling the identification of informal learning moments that may have remained hidden in traditional evaluation methods and provided insight into how participants translate learning outcomes into clinical practice. Future research on the effects of workplace-based interventions could benefit, therefore, from employing collective evaluation methods such as the effects forum. 

### Strengths and limitations

A strength of this study is the fact that the different stakeholders of the IntraPC learning activity were questioned collectively as opposed to individually. The use of a timeline was helpful to limit recall bias as well as to identify both formal and informal meaningful learning moments in daily activities. The collective approach offered participants the opportunity to gain deeper insights into IntraPC and helped bridge the gap between learning and practice by bringing stakeholders together to exchange perspectives.

An important strength of this study is that patients were included in the evaluation process. Residents mentioned that they particularly appreciated their presence, as patients could provide their perspective on whether behavioral changes had led to improved care. The inclusion of patients also provided important additional insights into their perspective on care across the PC–SC interface. Patients highlighted that their expectations of specialist care do not always align with the assumptions of referring primary care physicians, underscoring the importance of explicitly exploring and discussing these expectations, particularly when discrepancies arise. Moreover, patients offered concrete reflections on how residents’ collaborative behavior was experienced in practice. For example, they described how specific communication approaches contributed to them feeling more heard and involved in their care. In addition, patients made visible how little insight they typically have into the collaboration between primary and secondary care providers, and how greater transparency and communication about this process were highly valued. These contributions helped to surface aspects of IntraPC that often remain implicit for healthcare professionals, thereby contributing to co-construction on preferred IntraPC practices from the patient perspective.

During the effects forum, it was a difficult but essential taskto ensure that all voices were heard equally and could contribute equally. Patients reported that they found it difficult to follow discussions about SPICE formal learning activities or care structures across the PC-SC interface, mentioning that this difficulty could have hindered their ability to engage with certain technical statements regarding IntraPC across the PC-SC interface made by residents or supervisors. These findings align with previous studies, in which patients have expressed that they often experienced care across PC-SC interface as a ‘black box’, as something they were unaware of and not involved in, and that its occurrence and its quality were deduced from their appointments and contacts with their doctors and the actions they undertook [46].

A limitation to this study, therefore, may be that patients may not always have been fully able to participate in some discussions. To mitigate this as much as possible, one researcher closely monitored the participants (during the video conference), with particular attention to the patients, and occasionally provided clarification or paraphrasing to support their understanding and engagement. To facilitate patients in voicing their perspective, it could be beneficial in future evaluations to provide patients with more detailed preparation regarding the effects forum, PC-SC collaboration and the learning activities, which would allow them to articulate more confidently how the residents’ practices affected their care.

Conducting the effects forum in a hybrid format may have made it more difficult for some participants to respond.

Another consideration regarding dynamics between participants and researchers, is the dual role of the effects master. In both effects forums a resident came forward as the effects master. As a result of the presence of supervisors (including researcher MV), this may have limited their willingness to create memos on unintended or ‘negative’ outcomes. Second effects master and researcher WKS supported the resident effects master by initiating discussions on potential unintended outcomes, which led to the exploration of learning opportunities with potential ‘negative’ outcomes.

An important characteristic of this study is that the findings are strongly grounded in the specific context in which the research was conducted. The studied geriatric department is characterized by the provision of care for older patients with multimorbidity, in which close collaboration across the PC–SC interface is essential. Within this setting, attention to IntraPC has already been well established. This context offers rich opportunities for learning from PC–SC interactions. However, this context is already oriented towards integration and coordination across medical specialties. As a result, it is likely that not only the SPICE intervention, but also the existing workplace culture, contributed to the reported learning experiences. This is also reflected by the presence of daily interactions on our timeline of valuable IntraPC learning moments. Based on the available data, it is not possible to disentangle the influence of the intervention from that of the surrounding context. Furthermore, the study’s limited sample size (17 participants) and single-region focus may also restrict transferability. Consequently, the transferability of the findings to other settings is not self-evident. However, the insights generated in this study may serve as sensitizing concepts for future research and practice in different contexts. Nonetheless, the goal of this study was not to facilitate broad generalization but to provide a rich, contextualized understanding of the effects of SPICE, the complexity of evaluating workplace learning interventions and the potential of collective evaluation.

## Conclusion

The SPICE program on IntraPC across the PC-SC interface fostered insight into the care continuum, improved mutual understanding with actionable awareness and initiated a ripple effect of IntraPC learning across clinical settings. By way of this collective evaluation, participants revisited and deepened their learning in ways that align with the social and constructivist learning principles embedded in the program itself. This illustrates how collective evaluation can be a meaningful learning experience in its own right. When designing workplace-based learning interventions, incorporating evaluation methods that reflect social interaction as a learning opportunity may be valuable, therefore, not only to demonstrate their effects on clinical practice, but also to deepen and sustain the participants’ learning.

## Supplementary Information

Supplementary Material 1.

Supplementary Material 2.

## Data Availability

Upon reasonable request.
