# Supplementary material for: ‘A ripple effect of learning’: evaluating intraprofessional workplace learning in postgraduate medical education through collective evaluation
Source: BMC Med Educ. 2026 Jun 23;26:1422. doi: 10.1186/s12909-026-09777-5 (PMC13543373; doi:10.1186/s12909-026-09777-5)
Supplement: Supplementary file 1 [file 12909_2026_9777_MOESM1_ESM.docx]

# Appendix 1: The Secondary Primary care Intraprofessional Collaboration Education (SPICE) program

#### Aim

Primary care (PC) and secondary care (SC) residents learn intraprofessional collaboration(IntraPC) together during their co-location at the department of geriatric medicine of the Radboudumc in order to improve their collaborative practice and provide more coordinated and integrated care for frail older adult patients.

#### Design of SPICE

SPICE was created through Design Based Research by a team of educationalists, researchers, residents and supervisors, who combined IntraPC competencies and design principles (table 1 and 2) with boundary crossing theory (box theoretical background)

Each activity uses boundary activities between PC and SC, such as referral or hospital discharge, boundary objects, such as letters or phone calls, or boundary workers, such as PC residents in the patient journey of frail older adults. The formal learning activities are taught every month in a three-month cycle by a teacher with expertise in interdisciplinary education.

#### Curriculum

SPICE is a 3-6 month program that is taught throughout the entire year, consisting of four formal learning activities and two workplace learning activities (table 3). Residents enter SPICE upon the start of their rotations in geriatric medicine. SPICE takes three to six months depending on the duration of the resident’s rotation in geriatric medicine: three months for residents in internal medicine and general practice, and six months for residents in geriatric medicine and elderly care.

Every month, one of the formal learning activities occurs during the regular teaching hours in the workplace. The three activities for residents repeat every three months. Every week, residents are reminded of workplace learning opportunities during SPICE Tuesday.

Before the start of SPICE, supervisors in the geriatric medicine department were introduced to the topic of IntraPC through an introduction for supervisors.

Box: theoretical background – boundary crossing

# Theoretical background – Boundary Crossing

Boundary crossing theory is helpful in understanding IntraPC learning that may occur during PC and SC resident co-locations and can provide insight into how this learning may be improved. This theory describes the learning potential of working across boundaries. In this theory, boundaries are defined as sociocultural differences leading to discontinuity in action and interaction. An example of a boundary with significant learning potential is the PC-SC interface.

Boundary crossing occurs while working with boundary objects, boundary workers and boundary activities.

*Boundary objects* are physical artifacts that cross the boundary and have meaning on both sides.

*Boundary activities* are activities that occur around the boundary. In the case of the PC-SC interface,

*Boundary workers* are people that work across the boundary, ‘building bridges’ between the two sides.

In the case of the PC-SC interface, boundary objects could be referral or discharge letters, boundary activities are hospital admission or discharge and boundary workers could be PC residents during their SC geriatric medicine rotations.

In boundary crossing, four important learning mechanisms are described:

- In **identification**, learners gain insight into the roles, responsibilities and tasks involved in working on both their own and the other side of the boundary.
- In **coordination**, learners gain insight into the communicative connections across the boundary and learn ways to communicate across the boundary.
- In **reflection**, learners make and take perspectives on the processes of crossing the boundary, to gain a deeper understanding of the interactions taking place on both sides of the boundary.
- In **transformation**, learners collectively create new strategies for collaboration across the boundary, leading to a new shared collaborative practice

**Culture**

0. The patient is the starting-point for working and learning

1. Build relations with intraprofessional (primary–secondary care) colleagues

2. Apply the principle that, in a intraPC partnership, we are all different but operate on a basis of equity

**Connecting contexts**

3. Facilitate learning together by working together

4. Facilitate the acquisition of knowledge of one another's work contexts and activities to promote good collaboration.

**Making the implicit explicit**

5. Collaborate on patients and pay deliberate attention to two-way learning from different perspectives.

6. Purposely discuss intraPC collaboration during daily work activities.

7. Supervisors themselves engage in intraPC as role models.

8. The training team engages explicitly in intraPC with the aim of delivering quality patient care and achieving continuous quality improvement.

9. Bodies responsible for specialty programme goals define intraPC as a competency that *every* doctor should have.

10. Supervisors, teachers and residents work to ensure that every resident knows how to engage in intraPC upon completion of their training.

11. Residents transfer intraPC lessons and apply them in their own work contexts.

Competencies

Relationship building:

With patients and doctors

Transparent collaborating:

be able to provide clarity on the process of collaboration and on roles and responsibilities of those involved

Reflective practising:

To be willing to acknowledge mistake, give and receive feedback

**Table 1: IntraPC competencies**

**Table 2: IntraPC design principles and competencies**

**Table 3 Learning activities**

| **Learning activities** | **Goal** | **Design principles** |
| --- | --- | --- |
| **Formal learning activities** | |  |
| Kick Off | A meeting was held during regular teaching hours to introduce SPICE and get to know each other and each other’s specialty background. Ice breaking exercises were used to create a safe environment for IntraPC learning, such as:   - Two truths one lie about your specialty: residents discussed which of three statements about their specialty was false. - The prejudice game: residents discussed what were common prejudices about their specialty and reflected on the effect of prejudice on IntraPC. - Describing each other’s workday: SC residents tried to describe the workday of the PC residents and vice versa. - The imaginary line: residents ranked their specialty according to statements such as ‘other physicians are familiar with what my specialty’s responsibilities are’.   The activity ended with reflection on how the newly acquired knowledge about each specialty could impact IntraPC with this specialty. | 0  1  2  3  4 |
| Rich Pictures | In this session held during regular teaching hours**,** residents were asked to prepare a drawing on a topic related to IntraPC between PC and SC. Topics could include:  ‘What is your most/least successful PC/SC collaboration’  ‘What is important for PC/SC IntraPC’  Once every three months, this session is dedicated to the topic:  ‘What is important for a patient regarding PC/SC IntraPC’  Each drawing was then discussed during the formal learning activity. If participants could not explain a drawing, they tried to describe the drawing through the following steps:   1. What is depicted on the drawing? 2. What is the coherence between the elements on the drawing? 3. What did the drawer try to tell us through this drawing?   Then the drawer could clarify his/her drawing. The group discussed how the insights provided by the drawing could be translated to their own collaborative behaviour. | 0  1  2  3  11 |
| Patient Journey | In this meeting held during regular teaching hours, a PC and an SC resident prepared a presentation about a patient one of them had seen during their recent clinical work. Instead of focusing on the medical context of this patient, they described the journey this patient had made through PC and SC, using questions like:   - What moments of collaboration between physicians have occurred? - How did these physicians collaborate, through letters, over the phone etc.? - Was the collaboration sufficient or could it have been improved? - What can be learned about IntraPC from this patient journey?   The session ended with the residents reflecting on their own collaborative practice and formulating what they aimed to improve in their collaborative behaviour. | 0  3  4  10  11 |
| Introduction for supervisors | Firstly, a member of the design group provided an introductory presentation on the different formal and informal SPICE learning activities for PC and SC supervisors.  Secondly, the PC and SC supervisors familiarized themselves with the topic of IntraPC between PC and SC through a Rich Pictures session for supervisors only. | 0  3  7  8  9 |
| **Informal individual workplace learning activities** | |  |
| SPICE day | Each Tuesday during the morning handover, residents were encouraged to choose one moment in clinical practice in which they would pay dedicated attention to IntraPC. This could include:   - Carrying the referral phone - Discussing IntraPC aspects of referral/discharge letters - Partaking in joint consultation between a PC and an SC resident - Performing outpatient consultation triage.   At the end of the day, residents reflected on these activities with their supervisors. With their new insights, the resident and the supervisor together formulated what they aimed to improve in their collaborative behaviour. | 0  1  3  4  5  6  11 |
| Joint consultation in primary care | SC residents visited PC and worked together with PC physicians in their daily practice to get to know the PC context.  At the end of the day, the SC residents discussed the similarities and differences between their consultation styles with the PC supervisors or residents at the visiting location:   - What did they learn from the consultation? - Would the treatment plan made by SC be helpful for PC? - What would have happened if this consultation had occurred in PC?   The SC residents formulated how they aimed to improve their own consultation with these new insights. | 0  3  5  6  11 |
