# Supplementary material for: ‘A ripple effect of learning’: evaluating intraprofessional workplace learning in postgraduate medical education through collective evaluation
Source: BMC Med Educ. 2026 Jun 23;26:1422. doi: 10.1186/s12909-026-09777-5 (PMC13543373; doi:10.1186/s12909-026-09777-5)
Supplement: Supplementary file 2 [file 12909_2026_9777_MOESM2_ESM.docx]

# Appendix 3: interview guide ‘Effects forum’

**Welcome everyone,**

**Step 1: Introduction**

We are gathered here for an effects forum on the SPICE program. My name is Moderator 1; I am a geriatrician and a PhD candidate in the field of intraprofessional collaboration (IntraPC) learning. I am joined by Moderator 2 and Moderator 3, and we are grateful for your presence and participation in this effects forum.

Why are we conducting this effects forum? Healthcare is becoming increasingly complex, and collaboration among professionals is more essential than ever. Currently, care is often fragmented, with each professional developing their own treatment plan. For the future, we aim to achieve integrated care across primary and secondary care through intraprofessional collaboration. In such collaboration, all involved physicians jointly develop a shared care plan, with the patient at the center.

The SPICE program is designed to prepare future physicians for this approach. Since February 2021, we have implemented the “Secondary Primary Care Intraprofessional Collaboration Education” module, an educational program on collaboration for medical residents working in the Department of Geriatric Medicine at the Academic hospital XX in XX. This program specifically focuses on collaboration across the boundaries of the hospital, between primary care (PC) and secondary care (SC), for example, between general practitioners and geriatricians.

Today, we are convening an effects forum with participants from diverse backgrounds to explore the impact of this program on individual residents or supervisors, on interpersonal relationships, and on healthcare as a whole. We aim to do this by drawing on your experiences and narratives.

The effects forum is a method for engaging in dialogue, comparable to a focus group. All opinions and perspectives are welcome; there are no right or wrong answers. Your contributions will be used for a scientific publication; however, all data will be pseudonymized and cannot be traced back to you.

If you agree to participate under these conditions, I kindly ask you to sign the informed consent form and return it to me, or email it to me if you are participating digitally.

**Step 2: Check-in**

Before we begin, we will start with a brief check-in round. We will use the “popcorn method,” meaning you may contribute whenever a thought occurs to you rather than speaking in a fixed order. This round is solely for sharing; we will not respond to each other yet. We will spend approximately five minutes on this.

Before answering, please briefly introduce yourself by stating your name and professional background.

The question is:
“What do you consider important in intraprofessional collaboration across the PC–SC interface?”

**Step 3: Establishing the Timeline**

Now that everyone has checked in, we will begin the effects forum. The aim is to explore moments along a timeline during which you learned about IntraPC.

We will first identify these moments to construct a clear timeline, spanning from the start of your participation in SPICE until now. The predefined SPICE activities have already been placed on the timeline.

We are now interested in experiences that have stayed with you and in which you learned something about collaboration. These can include experiences during structured workplace learning activities (such as “SPICE tuesday” or joint consultation in PC), but also experiences from your personal life, patient encounters, or other relevant situations. There are no right or wrong answers.

**Step 4: Effects Master and Identifying Effects**

You will receive post-it notes and pens; digital participants have been provided with a link. We will now examine several of these moments in more detail.

If you believe you hear an effect being described, please write it down on a post-it or as a comment on the digital platform. I would like to ask one of you to take on the role of “effects master.”

As the effects master, if you identify an effect that you would like to explore further with the group, please signal this to me. We will then discuss collectively whether we indeed consider it an effect. You are also encouraged to ask follow-up questions if you wish to learn more about someone’s contribution.

Consensus is not required regarding the content of the effect, but agreement is needed on whether it constitutes an effect. Once the effect is clearly defined, we will write it on a post-it and place it on one of the posters: effects at the individual level, effects on interpersonal relationships, or effects on healthcare delivery. My colleague, Moderator 3, will assist you with this process.

**Step 6: Prioritizing Moments**

We will now select several of the identified moments for further discussion, following a chronological order. Which moments do you consider most important to prioritize?

**Step 7: Discussing Timeline Moments**

For each situation:

*Narrative*

What was the situation? Could you briefly describe it?

What was your role in this situation?

What happened? (Probe into context, cognitions, relationships, and system-level factors.)

*Actions*

What did you do, and why?

Who was involved? Do others recognize this situation?

*Effects*

What did you learn from this experience?

What has changed? (Probe into context, cognitions, relationships, and system-level factors.)

How do others perceive this?

**Step 8: Concluding the Timeline**

We are approaching the conclusion of the session. I would like to ask each of you to write down one effect and place it on one of the posters.

Could you briefly explain the effect you have identified?

**Step 9: Reflection on the Effects Forum Session**

In the final minutes, we will reflect on what you have learned from this session.

What do you take away from this session?

What will you do differently as a result?
